# Patterns of Public Interest in Lipomas and Lipoma-Removal Procedures: Google Trends Analysis

**DOI:** 10.2196/62993

**Published:** 2025-01-17

**Authors:** Keenan Duggal

**Affiliations:** 1 NYU Langone Health, 550 1st Ave, New York, NY, 10016, United States, 1 (212) 263-5290

**Keywords:** lipoma, fatty tumor, adipocyte, public interest, Google Trends

## Abstract

**Background:**

Lipomas are benign tumors composed of encapsulated adipocytes. Although relatively common, uncertainty remains about the population-level prevalence, the etiology, and the degree of public interest in lipomas and associated removal procedures.

**Objective:**

The spatiotemporal patterns of public interest in lipomas and lipoma removal procedures were characterized.

**Methods:**

Google Trends data that report the relative search volume (RSV) of Google queries pertaining to lipomas and their removal procedures at national and international levels were analyzed. To contextualize these trends, the RSV for lipomas was compared to that of several other common dermatological conditions in the United States.

**Results:**

In the United States, lipomas have consistently generated lower levels of public interest than other common dermatological conditions, but interest in the condition has been rising since the mid-2010s. Across the world, public interest in lipomas appears to be the highest in pockets of Eastern Europe, whereas in the United States, relative interest has been higher in Midwestern and Southern states. In addition, the interest in lipoma removal procedures has risen steadily from 2004 to the present, with particularly high RSVs coming from Southwestern states

**Conclusions:**

Dermatologists and plastic surgeons should be aware of the increasing public interest in lipomas and lipoma-removal procedures. Clinical awareness is especially important in states with an elevated interest in lipomas and their associated removal procedures.

## Introduction

Lipomas are benign adipocytic tumors [1,2]. While lipomas most commonly present on patients as solitary entities, it has been estimated that multiple lipomas develop in approximately 5% of affected patients [3]. In such cases, multiple fatty tumors can result from several rare medical disorders including familial multiple lipomatosis, Dercum disease, Madelung disease, and Gardner syndrome [1,4]. In general, the etiology of lipomas is unknown, but it has been postulated that soft-tissue injuries and genetic mutations might both play a role in their formation [5-8].

Lipomas can be unsightly to patients, can cause pain depending on their location [9,10], and can occasionally grow to sufficiently large sizes to disrupt quality of life [11,12]. Additionally, lipomas can have clinical presentations similar to more serious malignant liposarcoma tumors [13,14]. For these reasons, patients sometimes elect to have their lipoma(s) removed and biopsied. Generally, removal proceeds through intralesional injections of lipolytic agents, localized liposuction, or surgical excision [1,4].

While lipomas are estimated to affect around 1% of the population [1], the precise prevalence is difficult to estimate due to the elective nature of treatment and the nonexistence of universal screening modalities [15]. Compounding this uncertainty, very little is known about the degree of public awareness of lipomas and interest in associated treatments. These knowledge deficits restrict healthcare providers’ holistic understanding of the condition [16], which in turn might impede their ability to communicate with affected patients. To mitigate these knowledge gaps, Google Trends data were analyzed for the topic “Lipoma” and the query “Lipoma Removal” across time and several geographic scales. Because Google captures the vast majority of search engine traffic, the data provide a reasonable proxy for the totality of online public interest [17].

## Methods

To characterize public interest in lipomas, Google Trends data describing the national (United States) and international relative search volume (RSV) for the medical condition “Lipoma” between January 1, 2004 and May 21, 2024 were downloaded. Google Trends RSV data range from 0 to 100 and describe the relative frequency of a Google search, normalized to account for underlying spatiotemporal variation in internet usage. By using the topic “Lipoma,” the reported RSV reflects an aggregate value for all of the searched keywords/terms that “share the same concept in any language” [18]. In addition to temporal patterns, geographic patterns in lipoma-related searches were also assessed.

To situate public interest in “lipomas” within its broader dermatological context, the Google Trends “compare” feature was used to juxtapose the RSV of the topic “lipoma” against that of 4 other common dermatological conditions (queried equivalently as topics), which were selected to capture a range of prevalent skin conditions that can prompt dermatological consultation: atopic dermatitis, psoriasis, skin cancer, and rosacea [19,20].

To track public interest pertaining specifically to the “treatment” of lipomas, temporal and geographic trends were assessed using the search query “Lipoma removal.” I first verified that this query captures relevant public interest by analyzing the lists of “Related Topics” and “Related Queries.” For this search, two temporal windows were used for geographic comparisons: a long-term summary (January 1, 2004 to May 21, 2024) and a short-term summary (May 21, 2019 to May 21, 2024).

## Results

**Figure 1.**
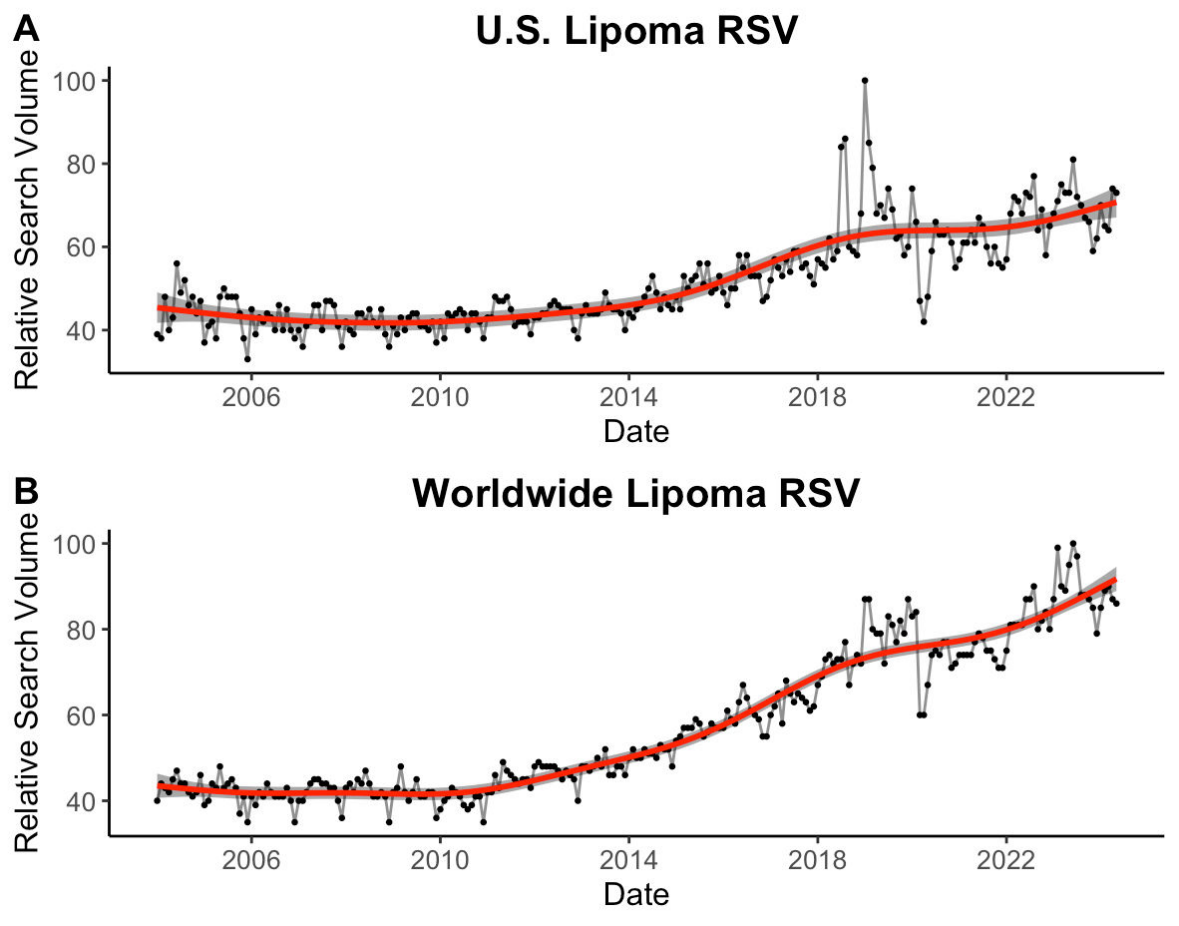
Temporal trends in public interest in lipomas. The monthly relative search volume (RSV) of lipoma-related search queries made on Google (A) in the United States and (B) around the world between January 1, 2004, and May 21, 2024, are plotted with generalized additive models and associated confidence intervals overlaid.

Over the past 20 years, there has been a substantial increase in public interest in lipomas in the United States and across the globe (Figure 1). Evanescent spikes in these time series are likely the product of transient popular cultural coverage related to the condition and/or significantly disruptive societal events (eg, the COVID-19 pandemic).

**Figure 2.**
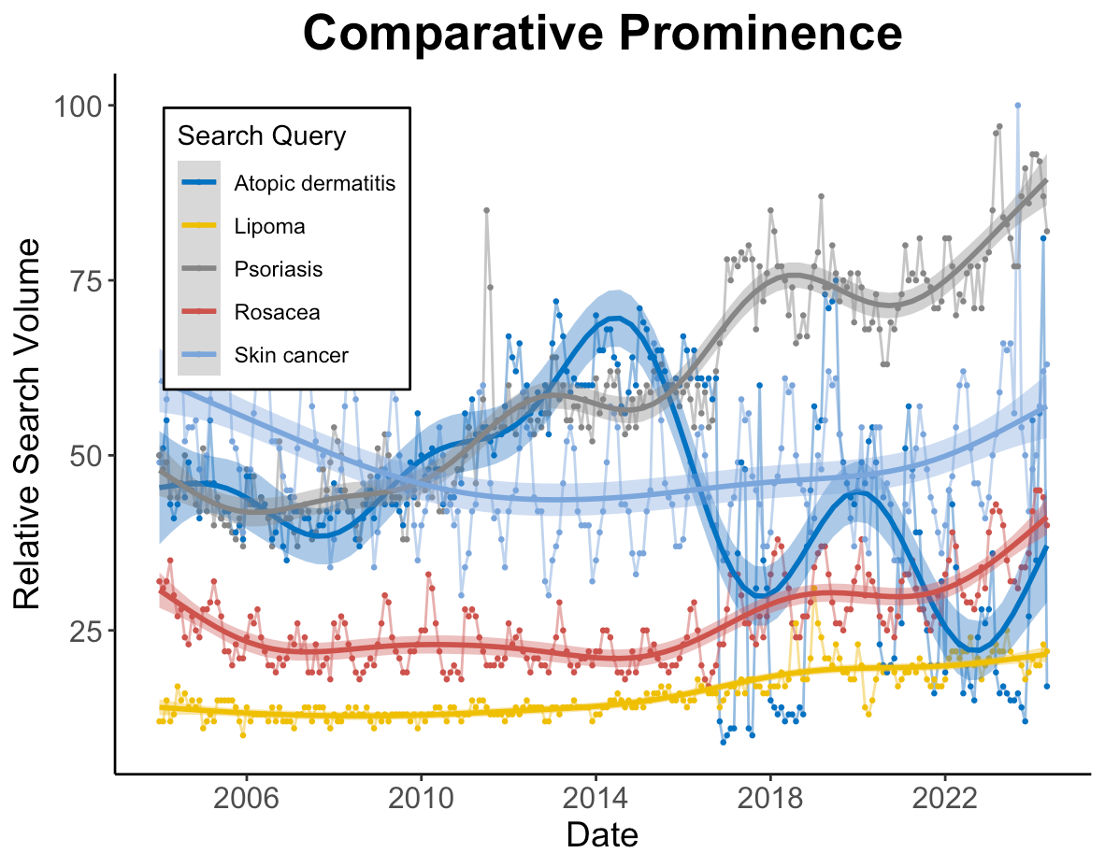
Comparative public interest in dermatological conditions. The Google Trends relative search volumes (RSVs) between January 1, 2004 and May 21, 2024 for 5 dermatological conditions are plotted on the same scale. Generalized additive models and associated confidence intervals describe smoothed trends over time.

Over the past 20 years, public interest in lipomas was consistently lower than that in 4 other common dermatological conditions: atopic dermatitis, psoriasis, skin cancer, and rosacea (Figure 2).

**Figure 3.**
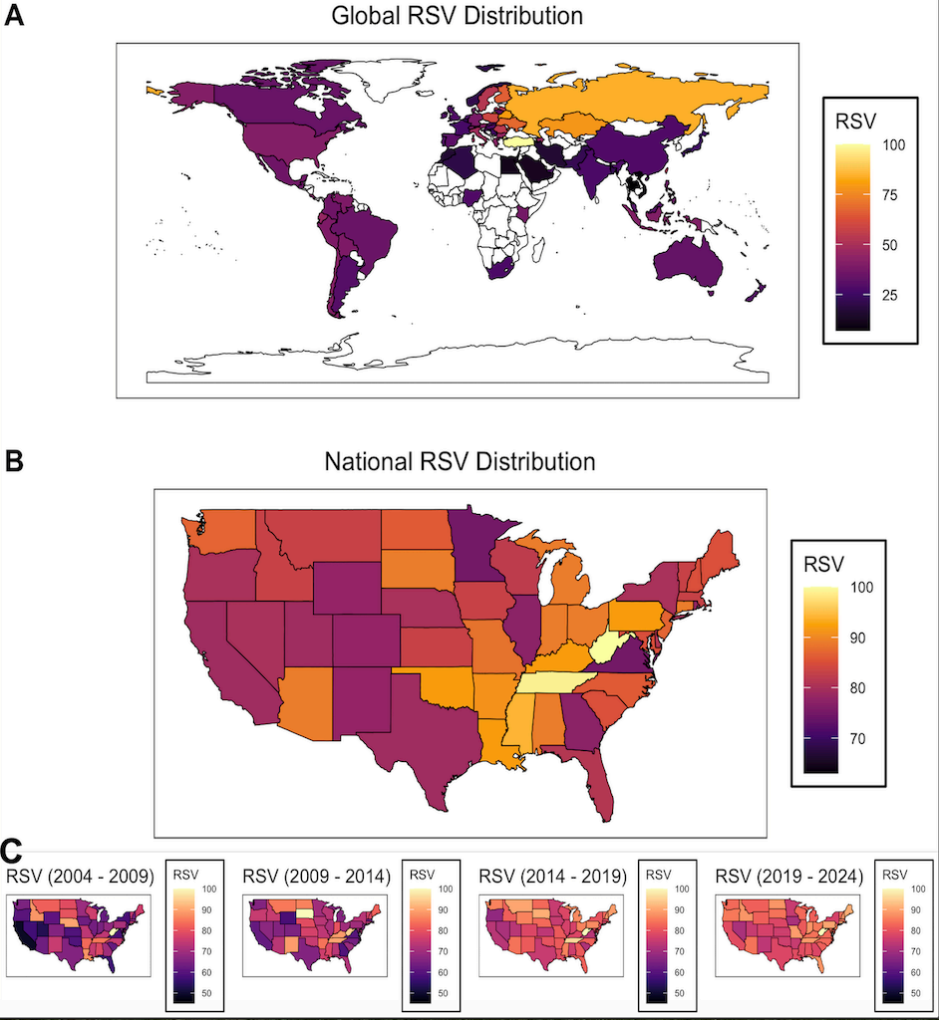
Geographic patterns of public interest in lipomas. (A-B) The relative search volume (RSV) of the Google search topic “lipoma” was compared across (A) countries and (B) contiguous states in the United States and averaged over the temporal period of January 1, 2004 to May 21, 2024. (C-F) The geographic distribution of “lipoma” RSV was compared in 5-year intervals in the contiguous states of the United States.

To characterize geographic regions with relatively elevated long-term interest in lipomas, the 20-year average country-level RSVs for the lipoma topic were examined. Lipoma-associated interest was the highest in pockets of Eastern Europe and Asia (Figure 3A). Specifically, the countries with the highest RSV for the lipoma condition were Turkey, Russia, Belarus, Kazakhstan, and Ukraine, with RSVs of 100, 84, 80, 78, and 68, respectively. Comparatively, the United States had an RSV of 41 for the same query.

Next, average state-level RSVs over the same period were assessed in the United States. Here, public interest in lipomas was the highest in Southern and Midwestern states (Figure 3B). Specifically, West Virginia, Tennessee, Mississippi, Kentucky, Pennsylvania, Louisiana, and Oklahoma each had RSVs greater than 92.

Finally, to evaluate if and how the geographic patterns of interest have changed over time in the United States, the average RSV of Google’s lipoma topic was assessed over 5-year increments from January 1, 2004, to January 1, 2024. While the spatial distribution of RSV remained relatively stable, the regional homogeneity generally increased over time (Figure 3C-F).

**Figure 4.**
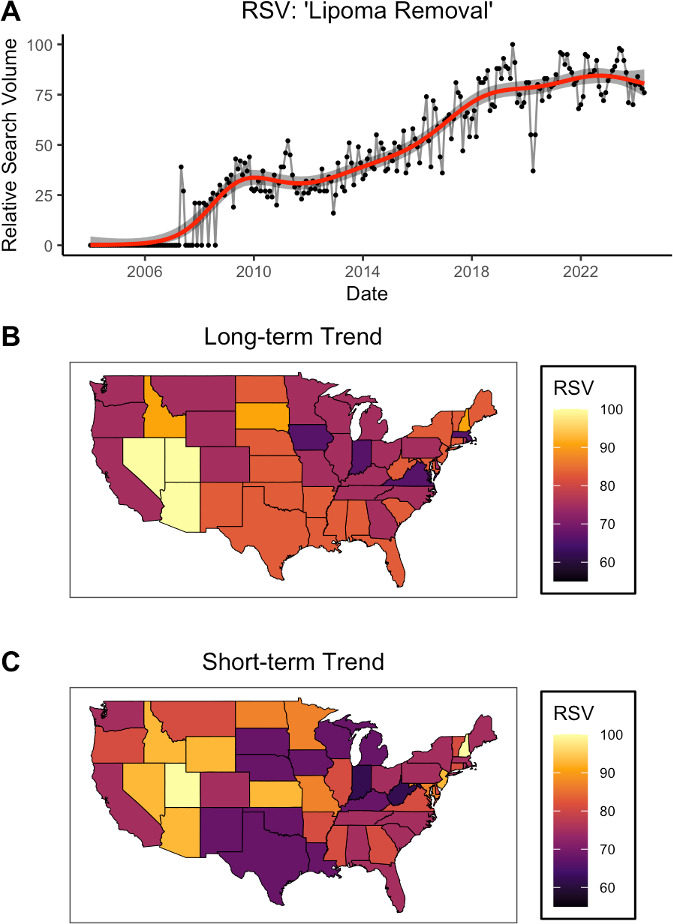
Public interest in lipoma removal (A) 20-year trend of monthly elative search volume (RSV) values for the Google search query “lipoma removal”. (B) Geographic distribution over the period of January 01, 2004 to May 21, 2024. (C) Geographic distribution over the period of May 21, 2019 to May 21, 2024.

There was a clear and consistent increase in Google searches containing both of the terms “lipoma” and “removal” from the mid-2000s onwards, interrupted only by a transient decline during the COVID-19 pandemic (Figure 4A). On average, interest in lipoma removal has been relatively higher in non-coastal Western states (Figure 4B). This long-term spatial pattern is largely consistent with that of the most recent 5-year period (Figure 4C).

## Discussion

This study leveraged Google Trends data to show that public interest in lipomas and lipoma-associated topics, while comparatively lower than that for other common dermatological conditions, has increased both in the United States and internationally over the past 20 years. Additionally, the interest in lipoma removal procedures has increased substantially over the past 20 years in the United States. While the descriptive nature of the analyses and claims presented here makes Google Trends a reasonable and sufficient source of data, the limitations inherent to Google Trends data must still be recognized (for instance that keyword selection can introduce bias, and that the RSV does not reflect the total search volume) [16,18,21].

While increasing interest in lipomas and lipoma-removal procedures could be explained by an increasing prevalence of the condition, consistent with increasing population-level adiposity [22], it is more likely that it is the result of increasing online health information-seeking behavior and increasing public health literacy [23,24]. Irrespective of the cause, increasing public interest in lipomas indicates a need for clinicians to be vigilant, prepared to encounter the condition, and comfortable with educating their patients about it.

The decrease in public interest in lipomas and associated “treatments” observed during the COVID-19 pandemic is reflective of a larger phenomenon wherein elective/aesthetic surgeries declined in popularity [25]. While it is logical that online interest in lipomas stalled during a period when other health concerns became more salient, it is surprising that there was no dramatic surge in interest in lipomas and lipoma-removal procedures, which was observed for similar cosmetic conditions and procedures, following the relaxation of public health guidelines [26]. Moving forward, it will be valuable to continue to monitor public interest relating to lipomas and other dermatological conditions to assess future trends and ultimately to inform healthcare practitioners of relevant patient interest [16,21].

## Abbreviations

RSV: relative search volume
